# Supplementary material for: Top-down beta oscillatory signaling conveys behavioral context in early visual cortex
Source: Sci Rep. 2018 May 3;8:6991. doi: 10.1038/s41598-018-25267-1 (PMC5934398; doi:10.1038/s41598-018-25267-1)
Supplement: Supplementary file 1 — Supplementary Figure S1 [file 41598_2018_25267_MOESM1_ESM.docx]

Top-down beta oscillatory signaling conveys behavioral context in early visual cortex

Craig G. Richter^1,2,3^, Richard Coppola^4^, Steven L. Bressler^1,5^

^1^Center for Complex Systems and Brain Sciences, Florida Atlantic University, 777 Glades Road, Boca Raton, FL 33431, USA

^2^Ernst Strüngmann Institute (ESI) for Neuroscience in Cooperation with Max Planck Society, 46 Deutschordenstrasse, 60528 Frankfurt, Germany

^3^BCBL. Basque Center on Cognition, Brain and Language, Mikeletegi Pasealekua 69, 20009 Donostia, Spain

^4^MEG Core Facility, National Institute of Mental Health, Bldg. 10, Rm. 4S235, 9000 Rockville Pike, Bethesda, MD 20892, USA

^5^Department of Psychology, Florida Atlantic University, 777 Glades Road, Boca Raton, FL 33431, USA

Correspondence should be addressed to Dr. Craig Richter, BCBL. Basque Center on Cognition, Brain and Language, Mikeletegi Pasealekua 69, 20009 Donostia, Spain or Dr. Steven Bressler, Center for Complex Systems and Brain Sciences, Florida Atlantic University, 777 Glades Road, Boca Raton, FL 33431, USA. E-mails: craiggrichter@gmail.com, bressler@fau.edu.
